# A biophysically grounded model of glutamatergic synaptic transmission integrating glutamate transport, receptor kinetics, and electrotonic effects

**DOI:** 10.1007/s10827-026-00935-8

**Published:** 2026-05-13

**Authors:** A. V. Chizhov, S. L. Malkin, D. N. Khachatryan, A. V. Zaitsev

**Affiliations:** 1 Computational Neurophysics Laboratory, Institute for Theoretical Physics, University of Bremen, Bibliothekstrasse 1, 28359 Bremen, Germany; 2 Sechenov Institute of Evolutionary Physiology and Biochemistry, Saint Petersburg, Russia; 3 Quantum Brains, Meraba Aleksidze street 12, 0171 Tbilisi, Georgia

**Keywords:** Glutamatergic transmission, Computational model, EAAT2 transporters, AMPA receptors, NMDA receptors, Synaptic plasticity, Electrotonic effects

## Abstract

Gluatamatergic synaptic transmission, critical for learning and memory, relies on precise regulation of extracellular glutamate levels by astrocytic transporters, particularly EAAT2. While existing models of AMPA and NMDA receptor kinetics often oversimplify glutamate dynamics or become computationally intractable, this study develops a balanced, biophysically grounded model that integrates glutamate transport, receptor sensitivity, and electrotonic effects. Using rat hippocampal slices, we recorded postsynaptic currents in CA1 pyramidal neurons under control conditions and during glutamate transporter blockade. The proposed mathematical model, formulated as a system of seven ordinary differential equations, distinguishes somatic and dendritic compartments, synaptic plasticity, and differential glutamate sensitivity of AMPA and NMDA receptors. Key findings reveal that the glutamate transporter blockade prolongs NMDA receptor-mediated currents without altering AMPA receptor kinetics, consistent with the higher glutamate sensitivity of NMDA receptors. The model also predicts glutamate concentrations in synaptic and extrasynaptic spaces, offering insights into spatial neurotransmitter dynamics. Furthermore, it accounts for voltage-dependent NMDA responses and short-term plasticity observed experimentally. By bridging the gap between oversimplified and overly complex approaches, this work provides a versatile tool for studying synaptic transmission in normal and pathological conditions, such as epilepsy or neurodegenerative diseases, where glutamate dysregulation plays a central role.

## Introduction

Glutamate, the primary excitatory neurotransmitter in the central nervous system, plays a pivotal role in synaptic transmission, plasticity, and learning (Meldrum, 2000). It mediates fast excitatory signaling through ionotropic receptors (Hansen et al., 2021), including AMPA and NMDA receptors, which are essential for information processing and memory formation (Reiner & Levitz, 2018; Riedel et al., 2003). The precise regulation of glutamate levels in the synaptic cleft and extracellular space is critical for maintaining the balance between excitation and inhibition, a process largely governed by astrocytic glutamate transporters, particularly EAAT2 (Chen et al., 2004; Scimemi, 2019; Tzingounis & Wadiche, 2007). Dysregulation of glutamate signaling is implicated in numerous neurological disorders, such as epilepsy, stroke, and neurodegenerative diseases, underscoring its importance as a focus of neuroscience research (Mogoanta et al., 2014; Pajarillo et al., 2019; Tanaka et al., 1997; Tian et al., 2005; Zaitsev et al., 2020).

Understanding the mechanisms underlying glutamatergic transmission is fundamental not only for elucidating normal brain function but also for uncovering the pathological processes underlying neurological diseases. Mathematical modeling has emerged as a powerful tool for studying these complex processes, enabling researchers to integrate experimental data and predict system behavior under various conditions. By simulating the dynamics of glutamate release, diffusion, and uptake, models can provide insights into how synaptic responses are shaped by factors such as receptor kinetics, electrotonic effects, and synaptic plasticity (Sylantyev et al., 2008, 2013; Tønnesen et al., 2018, 2023).

Existing models fall into two extremes: oversimplified approaches, or overly complex models that are computationally intractable. Here, we address this gap by developing a minimal yet biophysically grounded model that balances biological accuracy with computational efficiency. Simplistic models neglect the role of glutamate transport and diffusion. Excessively complex models incorporate detailed spatial dynamics of synaptic geometry that however render them computationally too expensive for large network simulations. Simplified models, typically based on systems of ordinary differential equations (ODEs), describe the opening and closing of AMPA and NMDA receptors but generally ignore the spatial aspects of glutamate diffusion and transport (Chizhov, 2014; Dayan & Abbott, 2001; Destexhe et al., 1994, 1998) . In such models without detailed consideration of neurotransmitter dynamics, the postsynaptic conductance is found as a solution of an ODE that filters an arbitrary presynaptic spike train or a firing rate with a one- or two-exponential time kernel. For example, in Chizhov (2014) a second-order ODE approximates the synaptic conductance as the convolution of a firing rate with the alpha function that shapes a single postsynaptic event (Rall, 1967). While these models are computationally efficient and useful for simulating network activity, they fail to capture the full complexity of synaptic transmission, particularly the role of glutamate clearance by astrocytes. Conversely, more complex models, which include detailed descriptions of synaptic geometry and molecular diffusion (Allam et al., 2012; Franks et al., 2002; Rusakov & Kullmann, 1998; Sylantyev et al., 2008, 2013), provide a more accurate representation of glutamate dynamics but are often computationally intensive and difficult to apply to large-scale simulations. This dichotomy highlights the need for a model that balances biological accuracy with computational tractability.

Here, we address this gap by developing a minimal yet biophysically grounded model of glutamatergic currents. The model incorporates (1) glutamate diffusion across synaptic and extrasynaptic compartments, (2) EAAT2-mediated uptake, (3) voltage-dependent NMDA receptor modulation, (4) short-term plasticity, and (5) electrotonic signal propagation in a two-compartment neuron. Formulated as a system of seven ODEs, the model balances biological fidelity with computational efficiency. We validate it against experimental recordings from rat hippocampal slices, demonstrating its ability to replicate key phenomena, including the differential effects of EAAT2 blockade on AMPA and NMDA receptor kinetics. Furthermore, the model predicts spatiotemporal glutamate gradients in the synaptic cleft—a variable challenging to measure experimentally.

The model is designed to describe the population-averaged response of the many synapses co-activated by Schaffer collateral field stimulation, rather than the response of a single synaptic contact; this population-level description is what makes it directly applicable to network-scale simulations. By unifying transporter dynamics, receptor sensitivity, and dendritic integration, this work advances our capacity to simulate synaptic transmission in health and disease, offering a versatile tool for both mechanistic studies and network-level explorations.

## Methods

### Experimental methods

#### Animal preparation and slice preparation

Experiments were performed on three-week-old male Wistar rats obtained from the animal facility of the Sechenov Institute of Evolutionary Physiology and Biochemistry, Russian Academy of Sciences (Saint Petersburg, Russia). Animals were housed under standard laboratory conditions with ad libitum access to food and water. All experimental procedures complied with the European Community Council Directive 86/609/EEC and were conducted in accordance with the institutional guidelines for the ethical use of laboratory animals.

Rats were euthanized by decapitation, and their brains were rapidly dissected. Brain slices were prepared as previously described (Postnikova et al., 2021). Briefly, horizontal slices (350 $\mu$m thick) were sectioned using a vibrating microtome (Microm HM 650 V, Microm, Germany). Slices were maintained in artificial cerebrospinal fluid (ACSF) with the following composition (in mM): 126 NaCl, 24 NaHCO$_3$, 2.5 KCl, 2 CaCl$_2$, 1.25 NaH$_2$PO$_4$, 1 MgSO$_4$, and 10 dextrose. The ACSF was continuously oxygenated with a mixture of 95% O$_2$ and 5% CO$_2$. Following sectioning, slices were transferred to oxygenated ACSF and incubated for one hour at 35 $^\circ$C before electrophysiological recordings. One to five slices from each rat were utilized for experiments.

#### Electrophysiological recordings

Whole-cell patch-clamp recordings were obtained from CA1 pyramidal neurons; a total of approximately 60 cells were recorded from 25 rats, with 4 representative neurons selected for model fitting. The neurons were additionally identified as principal excitatory cells by their regular spiking firing pattern, with prominent frequency adaptation within the train. For the recordings we used a pipette solution containing (in mM): 127 cesium methanesulfonate (CsMeSO$_4$), 10 NaCl, 5 EGTA, 10 HEPES, 6 QX314, 4 ATP-Mg, and 0.3 GTP, with the pH adjusted to 7.25 using CsOH, or a pipette solution containing (in mM): 136 potassium gluconate (K-Glu), 10 NaCl, 5 EGTA, 10 HEPES, 4 ATP-Mg, and 0.3 GTP, with the pH adjusted to 7.25 with KOH.. Series resistance was not compensated. Excitatory postsynaptic currents (EPSCs) were evoked by electrical stimulation of Schaffer collateral axons using a bipolar tungsten wire electrode. Recordings were acquired at 20-second intervals to ensure response stability.

AMPA receptor-mediated responses were recorded at a holding potential of -80 mV, while NMDA receptor-mediated responses were recorded at holding potentials of -20 mV and +40 mV. To isolate currents through AMPA and NMDA receptors, specific pharmacological agents were added to the perfusion solution: bicuculline (10 $\mu$M) to block GABA$_A$ receptors, DNQX (20 $\mu$M) to block AMPA receptors, and D-AP5 (50 $\mu$M) to block NMDA receptors. To examine the role of glutamate transport, the broad-spectrum EAATs inhibitor TFB-TBOA (300 nM), which blocks EAAT1, EAAT2, and EAAT3, was added to the external solution one hour prior to initiating electrophysiological recordings. Because EAAT2 is the predominant glutamate transporter at the hippocampal CA1 synapse and is expressed on astrocytes surrounding the synapse, we refer to this manipulation as “EAAT2-dominant blockade” throughout the manuscript; however, we acknowledge potential contributions from EAAT1 (astrocytic) and EAAT3 (postsynaptic neuronal) blockade. Statistical comparisons between control and TFB-TBOA conditions were performed using the two-tailed Student’s t-test (paired or unpaired, depending on the experimental design), with significance thresholds set at $p < 0.05$ (*), $p < 0.01$ (**), and $p < 0.001$ (***), as indicated in Fig. 4.

### Mathematical model

**Fig. 1:**
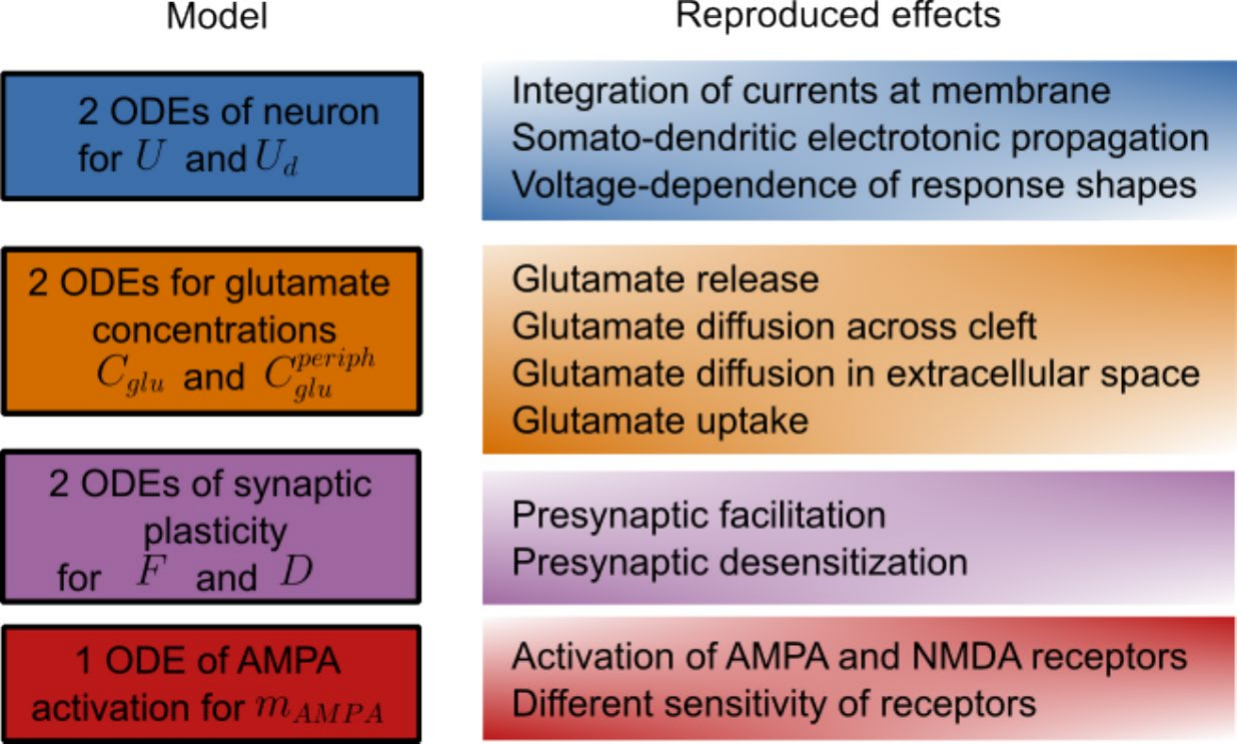
Schematic of the proposed model. The model is based on a system of seven ordinary differential equations (ODEs) and incorporates the key features listed above, enabling the reproduction of experimentally observed synaptic currents

We developed a mathematical model capable of reproducing experimentally observed synaptic currents, incorporating the following key features: (i) integration of currents at the membrane, (ii) somatodendritic electrotonic propagation, (iii) glutamate release, (iv) glutamate diffusion across the synaptic cleft, (v) voltage dependence of response shapes, (vi) glutamate diffusion between the synaptic cleft and the extrasynaptic space, (vii) glutamate uptake, (viii) presynaptic plasticity, including facilitation and desensitization/depression, and (ix) activation of AMPA and NMDA receptors, accounting for their differential sensitivity to glutamate (red and orange lines in Fig. 1). The proposed model is based on a system of seven ordinary differential equations (ODEs) and is designed to replicate the experimentally observed dynamics of synaptic transmission.

#### Neuron model

The mathematical model is based on a two-compartment neuron model (Chizhov et al., 2015), where both AMPA and NMDA receptors are assumed to be located on the dendrite. In the current-clamp (CC) mode, the model consists of two equations describing the dendritic and somatic membrane potentials:

1 $$\begin{aligned} \tau _{m}\frac{dU}{dt} = - \left( U - U_{L} \right) + \frac{2\gamma }{l}\left( U_{d} - U \right) + \frac{I_{inj}}{G_{s}} \end{aligned}$$

2 $$\begin{aligned} \tau _{m}\frac{dU_{d}}{dt} = - \left( U_{d} - U_{L} \right) - \frac{2}{l}\left( U_{d} - U \right) + \frac{I_{d}}{\gamma G_{s}} \end{aligned}$$

Here, $G_{s}$ is the soma conductance, $\gamma$ is the ratio of the membrane areas of the dendrite and soma, *l* is the square of the ratio of the dendrite length to the characteristic length $\lambda$, $\tau _{m}$ is the characteristic membrane time, $I_{inj}$ is the current injected at soma, and $I_{d}$ is the synaptic current received at the dendrite. The input conductance for the somatically injected current, derived from the steady-state solution of the above equations, is $G_{in} = G_{s}(3 + 2\gamma )/(3 + \gamma )$.

The synaptic current received by dendrites, $I_{d}(t)$, is either directly calculated from known AMPA and NMDA synaptic conductances or approximately reconstructed from the somatically registered current $I_{VC}(t)$. In the former case:

3 $$\begin{aligned} I_{d}(t) = I_{AMPA}(t) + I_{NMDA}(t) \end{aligned}$$

where

4 $$\begin{aligned} I_{AMPA}(t) = {\overline{g}}_{AMPA}\ m_{AMPA}(t)\ \left( U_{AMPA} - U(t) \right) , \end{aligned}$$

5 $$\begin{aligned} I_{NMDA}(t) = {\overline{g}}_{NMDA}\ m_{NMDA}(t)\ f_{NMDA}\left( U_{d}(t),{[Mg]_{o}} \right) \left( U_{NMDA} - U(t) \right) \end{aligned}$$

Here, $m_{AMPA}(t)$ and $m_{NMDA}(t)$ are receptor activation variables, ${\overline{g}}_{AMPA}$ and ${\overline{g}}_{NMDA}$ are scales for the conductances, and

6 $$\begin{aligned} f_{NMDA}\left( V,[Mg]_{o} \right) = {1}/{\left( 1 + \frac{[Mg]_{o}}{3.57}\exp ( - 0.062\ V) \right) } \end{aligned}$$

is the voltage-dependent factor of the magnesium blockade of NMDA channels (Jahr & Stevens, 1990).

In the case of $I_{d}(t)$ reconstructed from the somatically registered current $I_{VC}(t)$, it is approximated as in Chizhov et al. (2015):

7 $$\begin{aligned} I_{d}(t) = \left( \frac{l}{2}\tau _{m}\frac{d}{dt} + 1 + \frac{l}{2} \right) I_{VC}(t) \end{aligned}$$

In the voltage-clamp (VC) mode, with the somatic potential fixed at $U = V_{h}$, the dendritic voltage equation remains to be governed by Eq. (2); and its resting potential is $U_{d}^{0} = (U_{L} + 2V_{h}/l)/(1 + 2/l)$. The dendritic synaptic currents are expressed through synaptic conductances by Eqs. (3-6). The somatically recorded current $I_{VC}(t)$ is proportional to the voltage gradient and is given by:

8 $$\begin{aligned} I_{VC}(t) = \ 2(G_{s}\gamma /l)\ \left( U_{d} - U_{d}^{0} \right) \end{aligned}$$

#### Synaptic kinetics and sensitivity

**Fig. 2:**
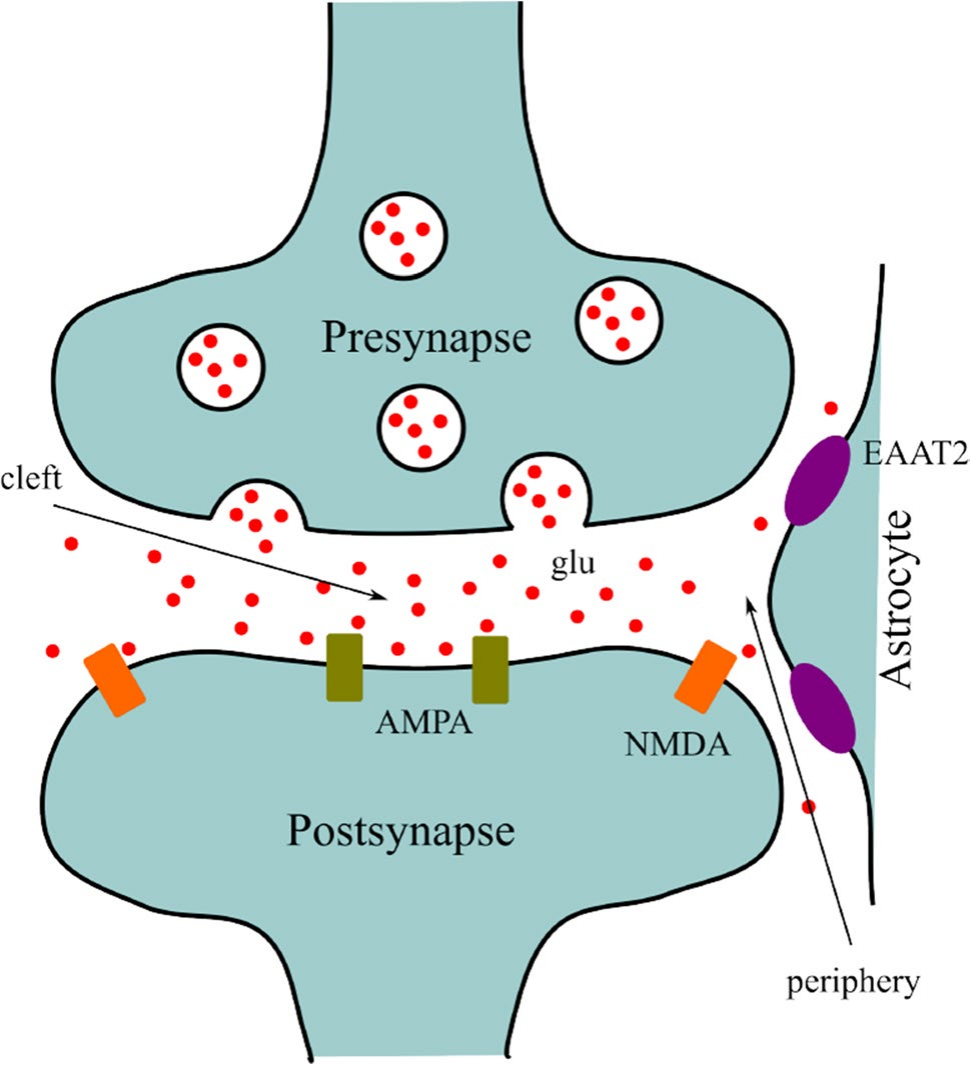
Synapse. Glutamate is released from the presynaptic terminal (top, blue), diffuses through the extracellular space from the synaptic cleft toward glial cells (right), where it is taken up by EAAT2 transporters

We distinguish two compartments of extracellular space: the intrasynaptic and extrasynaptic regions (Fig. 2). AMPA receptors are located exclusively within the synaptic cleft, while NMDA receptors are predominantly located outside the cleft.

The AMPA receptors are activated by glutamate, then they deactivate and desensitize. The activation is much faster than deactivation (Hansen et al., 2021; Silver et al., 1996) and mainly determined by the dynamics of glutamate concentration, whereas the deactivation is characterized by some time scale $\tau _{A}$. We describe the kinetics of AMPA receptor conductance by the first-order ODE:

9 $$\begin{aligned} \left( \tau _{A}\frac{d}{dt} + 1 \right) m_{AMPA}(t) = S_{AMPA}\left( C_{glu}(t) \right) {D(t)} \end{aligned}$$

where $\tau _{A}$ is the closing time of AMPA receptors, $S_{AMPA}$ is the AMPA receptor sensitivity, represented by a sigmoidal activation function dependent on the glutamate concentration in the central part of the synaptic cleft, $C_{glu}(t)$, and *D*(*t*) is the receptor desensitization factor.

In contrast, the kinetics of NMDA receptors is assumed to be instantaneous, with activation driven by the glutamate concentration at the periphery of the synaptic cleft, $C_{glu}^{periph}$ (Cavalier et al., 2005; Hansen et al., 2021). Thus, the synaptic conductance follows the glutamate concentration without delay:

10 $$\begin{aligned} m_{NMDA}(t) = S_{NMDA}\left( C_{glu}^{periph} \right) -S_{NMDA}\left( C_{glu}^{0} \right) \end{aligned}$$

where $S_{NMDA}$ is the NMDA receptor sensitivity, also represented by a sigmoidal activation function dependent on the glutamate concentration. Here $C_{glu}^{0}$ is the background glutamate concentration in the resting state. The term $S_{NMDA}\left( C_{glu}^{0} \right)$ evaluates the fraction of desensitized NMDA receptors by that constantly present glutamate.

Based on experimental data (Featherstone & Shippy, 2008), the activation functions $S_{AMPA}$ and $S_{NMDA}$ are approximated as follows:

11 $$\begin{aligned} S_{AMPA}(x) = \frac{1}{\left( 1 + \left( \frac{1000\mu M}{x} \right) ^{1.6} \right) },\quad S_{NMDA}(x) = \frac{1}{(1 + 4.7\mu M/x)} \end{aligned}$$

**Fig. 3:**
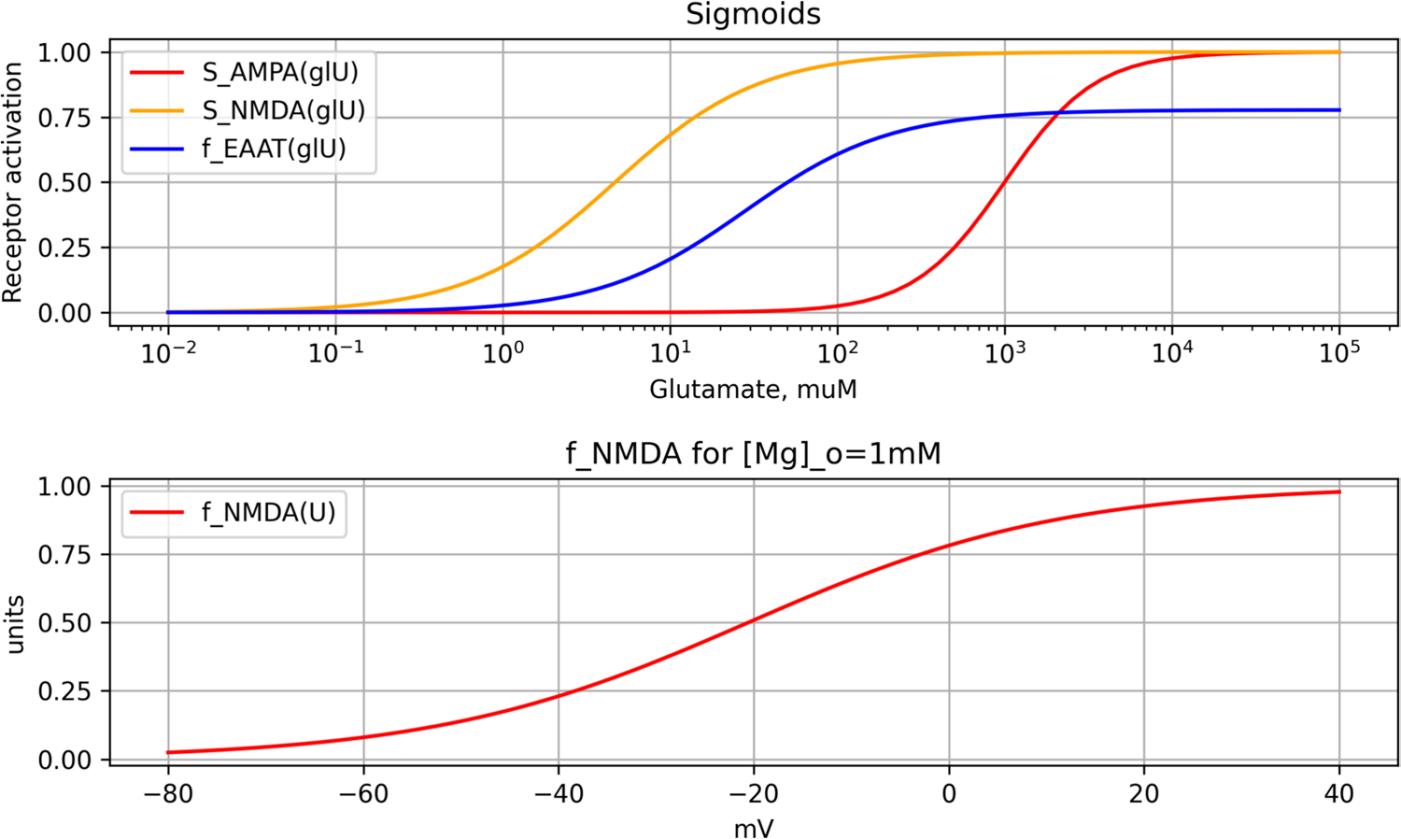
Sensitivity of AMPA and NMDA receptors to glutamate concentration. The activation functions $S_{AMPA}$ and $S_{NMDA}$ illustrate the differential sensitivity of AMPA and NMDA receptors to glutamate

These functions highlight the significantly higher sensitivity of NMDA receptors to glutamate compared to AMPA receptors (orange vs. red in Fig. 3).

#### Glutamate transport

When entering the synaptic cleft, glutamate affects AMPA receptors present there and further diffuses outwards, activating peri- and extrasynaptic NMDA receptors. The concentrations of glutamate in the central compartment of the synaptic cleft and its periphery (Hires et al., 2008) can be described by relaxation equations:

12 $$\begin{aligned} \frac{dC_{glu}}{dt} = \frac{C_{glu}^{periph} - C_{glu}}{\tau _{glu}}\ + W\ F(t)\ \nu (t) \end{aligned}$$

13 $$\begin{aligned} \frac{dC_{glu}^{periph}}{dt} = \frac{C_{glu} - C_{glu}^{periph}}{\beta \ \ \tau _{glu}} - \frac{ C_{glu}^{periph}-C_{glu}^{0}}{\tau _{diff}} - \alpha ^{periph}\ f_{EAAT2}(C_{glu}^{periph}) \end{aligned}$$

Here the first term in the right-hand side of Eq. (12) describes the diffusion of glutamate from the central synaptic cleft to the peripheral compartment. The second term in Eq. (12) is the release from the presynapse, where $\nu (t)$ is the synaptic pulse(s) of glutamate, evoked by a single spike of a series of spikes. In our simulations, $\nu (t)$ is shaped as square pulses characterized by the amplitude $\nu ^{pulse}$ and the duration $\Delta t^{pulse}$, where $\nu ^{pulse}$ is the flux of mediator reaching the receptors at postsynaptic membrane inside the cleft during the time period $\Delta t^{pulse}$. Note that the time of the glutamate diffusion through synaptic cleft is taken into account just implicitly through $\Delta t^{pulse}$. Thus, for instance, the change of the width of synaptic cleft affects both, $\nu ^{pulse}$ and $\Delta t^{pulse}$, such that the total mediator quantity $\nu ^{pulse} \cdot \Delta t^{pulse}$ is not affected; and the major expected effect is the reshaping of the rising phase of synaptic current. Notably, Equation (12) does not include an EAAT uptake term in the central cleft, reflecting the experimental and modelling evidence that EAAT2 transporters are predominantly located in the perisynaptic/extrasynaptic astrocytic membrane rather than within the synaptic cleft itself (Tzingounis & Wadiche, 2007).

The first term in the right-hand side of Eq. (13) describes diffusion from the peripheral compartment back toward the synaptic cleft. The second term in Eq. (13) is the diffusion between the peripheral compartment and the bulk of the neural tissue and the perfused solution where we denote the concentration as $C_{glu}^{0}$. In the control case of intact glutamate transporters, this background concentration is assumed to be zero, whereas it is not-negligible in the case of transporter blockage. The last term reflects the glutamate removal by the EAAT2 transporters, with the maximum rate $\alpha ^{periph}$. The glutamate transporter blockade is modelled by setting $\alpha ^{periph} = 0$.

The other parameters are: $\beta$ is the ratio of the volume of the extracellular space to the volume of the synaptic cleft, $\tau _{glu}$ is the characteristic time of diffusion from the synaptic cleft to the rest of the extracellular space, $\tau _{diff}$ is the characteristic time of glutamate diffusion from the extracellular space to the perfusion solution, *F*(*t*) and *D*(*t*) are the presynaptic facilitation and desensitization/depression factors, and *W* is the contribution of one spike to the change in mediator concentration. (For taking into account the electrodiffusion (Savtchenko et al., 2017; Sylantyev et al., 2013), we might consider the diffusion scale to be dependent on the holding voltage, $\tau _{glu}(V_{h})$, however, this generalisation was not needed for the reproduction of the experimental data and thus was avoided.)

EAAT2 transports glutamate molecules using the Na$^+$ ions’ gradient (Cater et al., 2016), according to the following formulae:

14 $$\begin{aligned} f_{EAAT2}(C_{glu}) = \frac{\left( Na^{e} - Na^{a} \right) ^{1.5}}{(50mM)^{1.5} + \left( Na^{e} - Na^{a} \right) ^{1.5}}\ \frac{C_{glu}}{k_{1/2}^{Glu} + C_{glu}} \end{aligned}$$

where $Na^{e} = 130mM$ is the extracellular sodium concentration, $Na^{a} = 15mM$ is the sodium concentration in the astrocyte, the sodium concentration required to achieve the half-maximum current is set to be 50mM, and $k_{1/2}^{Glu}$ is the glutamate concentration required to achieve the half-maximum.

#### Synaptic facilitation and desensitization

**Fig. 4:**
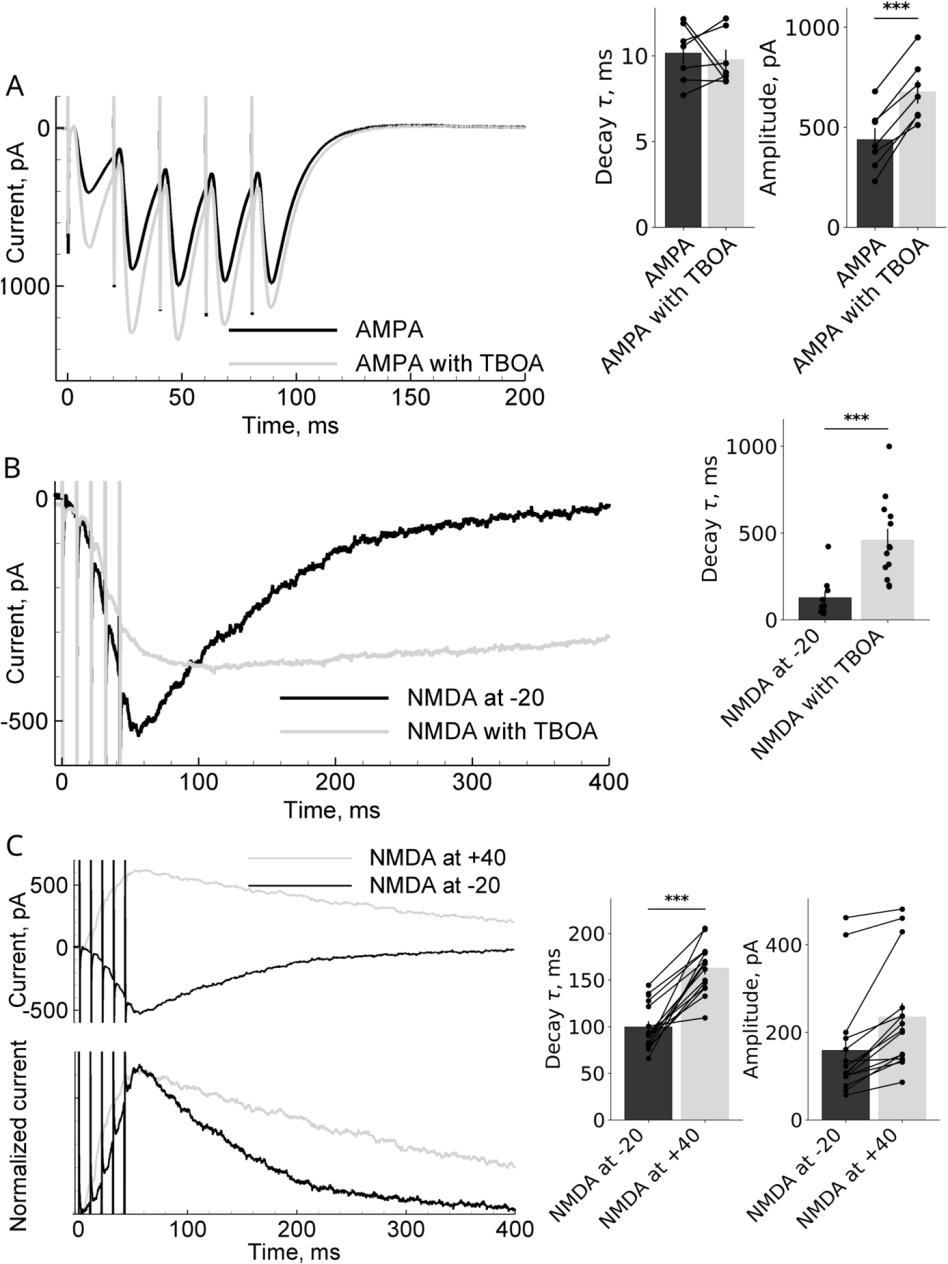
Experimental data from representative neurons. (**A**) AMPA receptor-mediated responses to a train of five stimuli under control conditions and in the presence of EAAT2 blocker TFB-TBOA, recorded at a holding potential of -20 mV. (**B**) NMDA receptor-mediated responses to a train of five stimuli under control conditions and in presence of TFB-TBOA, recorded at a holding potential of -20 mV. (**C**) Control NMDA receptor-mediated responses recorded at holding potentials of -20 mV and +40 mV. The normalized responses (below) reveal differences in shapes at -20 mV and +40 mV. Right panels show populational characteristics: decay time and amplitude. Each dot represents one recorded cell

Repeating synaptic stimulation changes the portion of released mediator and the efficacy of receptors (Hansen et al., 2021). We take these processes into account in the form of synaptic facilitation *F*(*t*) and desensitization of AMPA receptors *D*(*t*), thus neglecting the presynaptic depression in favor of prevailing desensitization (Chen et al., 2002). Naturally, the facilitation *F*(*t*) affects the glutamate concentration through Eq. (12), and the desensitization *D*(*t*) affects the AMPA conductance through Eq. (9). The equation for presynaptic facilitation *F*(*t*) was modified after the Tsodyks-Markram model (Markram et al., 1998). Whereas in their model the facilitation and depression depend on the timing of presynaptic spikes or the presynaptic firing rate, we experimentally observed that in response to a single short stimulus the AMPA receptor activation changes after the blockade of EAAT2 (compare gray to black lines in Fig. 4A). Therefore, we assume that *F*(*t*) depends on the glutamate concentration. This dependence may be explained by the following mechanism. The facilitation depends on the pool of synaptic vesicles ready for release. This pool depends on the calcium ions entering the presynapse through the voltage-gated synaptic channels. In its turn, the depolarisation is mediated by either the metabotropic glutamate receptors (Chu & Hablitz, 2000) or extrasynaptic NMDA receptors (Duguid & Smart, 2009). Thus, we assume that *F*(*t*) depends on the glutamate concentration in the periphery of the cleft, $C_{glu}^{periph}$, instead of the presynaptic spike rate:

15 $$\begin{aligned} \frac{dF}{dt} = \frac{F_{0} - F\ }{\tau _{F}} + H_{F}(1 - F)\ \frac{C_{glu}^{periph}(t)}{k_{1/2}^{Glu}} \end{aligned}$$

where $\tau _{F}$ is the characteristic time of facilitation decay; $F_{0}$ is the resting value of *F*(*t*); $H_{F}$ is the increase of *F* after each stimulus; $k_{1/2}^{Glu}$ is the scale for the concentration, taken from Eq. (14).

For the desensitization *D*(*t*), we modify the equation for presynaptic depression from the Tsodyks-Markram model, assuming that the process affect those AMPA receptors that were activated by the glutamate in the cleft, thus proportionally to $S_{AMPA}(C_{glu}(T))$:

16 $$\begin{aligned} \frac{dD}{dt} = \frac{(1 - D)\ }{\tau _{D}} - H_{D}D\ {S_{AMPA}\left( C_{glu}(t) \right) } \end{aligned}$$

where $\tau _D$ is the time scale of recovery from desensitization; and $H_{D}$ controls the decrease of receptor efficacy after each stimulus. Note that the Eqs. (9, 12, 15 and 16) imply that *F*(*t*) affects the glutamate concentration and thus both, AMPA and NMDA, conductances, whereas *D*(*t*) does the AMPA conductance only. The presynaptic depression may implicitly contribute through the parameters for *D*(*t*), thus it might be called as a desensitization/depression factor.

Finally, we obtain a system of seven first-order ODEs, where 2 ODEs describe the neuronal membrane polarization, 1 ODE approximates the synaptic kinetics, 2 ODEs describe the glutamate concentrations in two compartments, and 2 ODEs are for the synaptic plasticity.

### Numerical implementation

The model was implemented in Python (version 3.10), using the scipy.integrate.odeint function for the differential equation integration. Parameter fitting was performed by minimizing the mean-squared error between simulated and experimental traces using the Nelder-Mead method realized in scipy.integrate.minimize function, starting from physiologically plausible initial parameter values (see parameter table). The model code is available at https://github.com/antonvchizhov/EAAT-paper.git.

## Results

### Experimental observations

Pharmacologically isolated synaptic currents through AMPA and NMDA receptors were recorded in pyramidal cells of the CA1 region of the hippocampus in response to stimulation of Schaffer collaterals in rat brain slices. Stimulation was delivered every 20 seconds as a train of five pulses at a frequency of 50 Hz. Several key features of these responses were identified, presenting challenges for accurate replication in mathematical models.

#### Effect of EAAT2 blockade on receptor kinetics

Pharmacological blockade of EAAT2 transporters did not alter the kinetics of AMPA receptor-mediated currents (Fig. 4A). In contrast, the decay phase of NMDA receptor-mediated currents was significantly prolonged compared to control conditions (compare gray to black lines in Fig. 4B). Accurately describing this differential effect of EAAT2 blockade poses a critical challenge for the mathematical model.

#### Short-term synaptic plasticity in AMPA receptor-mediated currents

When recording AMPA receptor-mediated currents in response to a train of five stimuli, we observed changes in response amplitudes. The first response in the train exhibited the smallest amplitude compared to subsequent responses, indicating an initial phase of facilitation (Fig. 4A, black line). This phenomenon, characteristic of short-term synaptic plasticity, was incorporated into the mathematical model to provide a more accurate representation of synaptic transmission dynamics.

#### Voltage dependence of NMDA receptor-mediated currents

NMDA receptor-mediated responses recorded at holding potentials of -20 mV and +40 mV displayed similar amplitudes (Fig. 4C). This observation is notable because at -20 mV the driving force is stronger and a significant effect of voltage-dependent magnesium block might be expected, therefore the response at -20 mV is expected to be smaller than at +40 mV. Modeling was used to explore this apparent discrepancy. In contrast to the amplitudes, the shapes of the NMDA receptor-mediated responses at -20 mV and +40 mV differed significantly, as seen from comparison of normalized responses in Fig. 4C. We employed the model to investigate whether this difference could be attributed to electrodiffusion or other factors.

### Model fitting to experimental data

**Fig. 5:**
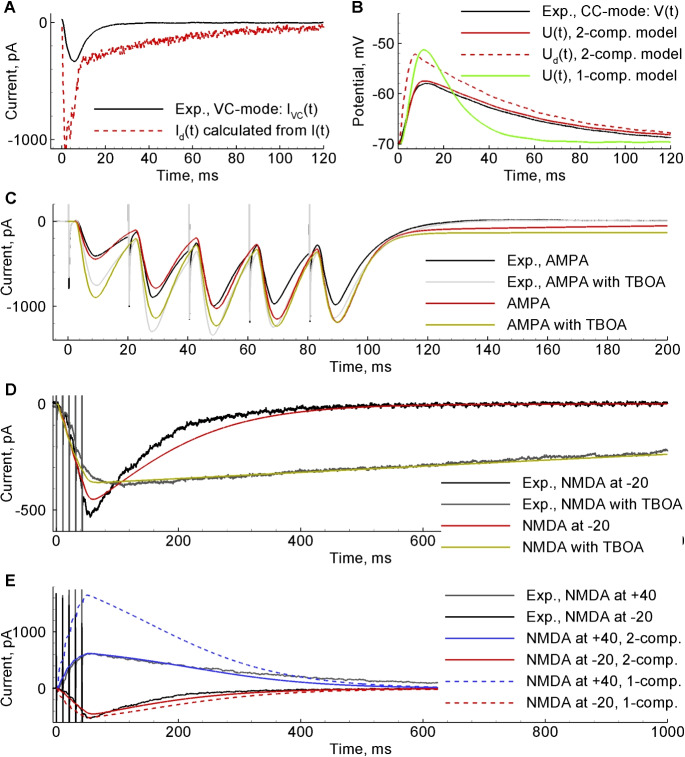
Fitting model to experiments. **A**, experimental current $I_{VC}(t)$ registered at soma of Cell-1 in VC-mode (black) and the dendritic current $I_{d}(t)$ calculated from $I_{VC}(t)$ with Eq. (7). **B**, experimental membrane potential in CC-mode, *V*(*t*), registered in Cell-1 in the same conditions (black); membrane potentials at soma, *U*(*t*) (red), and at dendrite, $U_{d}(t)$ (dotted), reconstructed in the 2-compartment model, Equations (1, 2), from $I_{d}(t)$; membrane potential in the 1-compartment model (green). **C**, response of Cell-2 to the series of 5 stimuli, registered at $V_{h} = - 80mV$ in control conditions (black) and in presence of glutamate transporter blocker TBOA (gray); modelled responses in control (red) and TBOA (green). **D**, response of Cell-3 to the series of 5 stimuli in presence of AMPA-receptor blocker registered at $V_{h} = - 20mV$ without (black) and similar response of Cell-4 with TBOA (gray); modelled responses (red and yellow, respectively). **E**, similar responses without TBOA (Cell-3) at $V_{h} = - 20mV$ (black) and $V_{h} = + 40mV$ (gray); modelled responses in 2-compartment model (red and blue, respectively) and in 1-compartment model (dotted lines)

To obtain the coefficients of the model, we use the set of representative experimental recordings shown in Fig. 5. Each type of response was recorded from approximately ten cells. We chose data from 4 representative neurons for fitting of the model parameters. Recordings in two of them were done in the CsMeSO$_4$ based solution and in another ones in the K-Glu based solution. For these neurons we estimated the input resistances (from measured input conductance $G_{in}$) which were then fixed during fitting; the rest of the parameters were fitted, assuming them to be the same for all 4 cells. The fitting procedure implied the solution of an optimization problem to minimize the mean squared difference between model and experimental traces, initialized from physiologically plausible parameter range. The fitting procedure was run in four steps: first, to find the parameters for the neuron model by reproducing the current-clamp based on voltage-clamp recordings; second, for the AMPA currents with and without TFB-TBOA application; third, for the NMDA currents; and finally, for the complete set of recordings to finally establish the entire parameter set. The final set of parameters is given in Table 1. The fitting converged robustly; sensitivity to initial conditions was explored by perturbing all parameters with random numbers dispersed within 50% and verifying that the optimal values were recovered with at least 20% precision. TFB-TBOA was implemented in the model by setting $\alpha ^{periph} = 0$ and non-zero $C_{glu}^{0}$, i.e., removing the EAAT2-mediated glutamate uptake term in Eq. (13).

**Table 1:** Model parameters. “Fitted” indicates values obtained by fitting to experimental data. “Literature” indicates values taken from published sources. “Fixed” indicates values set from direct experimental measurements

Parameter	Value	Source	Description
$\tau _m$	11 ms	Fitted	Membrane time constant
$\gamma$	2	Fitted	Dendrite/soma area ratio
*l*	1.3	Fitted	Electrotonic parameter
$G_S^{Cell1..4}$	8, 12, 8.2, 9 nS	Fixed (exp.)	Somatic conductance
$V_L^{Cell1..4}$	$-70, -69, -34, -4$ mV	Fixed (exp.)	Leak reversal potential
$W\nu ^{pulse}$	200 $\mu$M/ms	Fixed	Release amplitude for Eq. (12)
$\Delta t^{pulse}$	1 ms	Fixed	Stimulus pulse duration
$\overline{g}_{AMPA}$	3040 nS	Fitted	Scale for AMPA conductance
$\overline{g}_{NMDA}$	75 nS	Fitted	Scale for NMDA conductance
$[Mg]_o$	1 mM	Bezzi et al. (1998)	Extracellular Mg$^{2+}$
$U_{AMPA}$	0 mV	Literature	Reversal potential
$U_{NMDA}$	0 mV	Literature	Reversal potential
$\tau _{glu}$	2.9 ms	Fitted	Cleft diffusion time
$\tau _{diff}$	1.17 s	Fitted	Extrasynaptic diffusion time
$\beta$	66	Fitted	Volume ratio
$C_{glu}^0$	2.0 $\mu$M	Fitted	Background $C_{glu}$ in TBOA
$\alpha ^{periph}$	0.31 $\mu$M/ms	Fitted	EAAT uptake rate
$k_{1/2}^{Glu}$	28 $\mu$M	Cater et al. (2016)	EAAT half-max concentration
$\tau _F$	0.16 ms	Fitted	Facilitation decay time
$H_F$	64 ms$^{-1}$	Fitted	Facilitation increment
$F_0$	0.42	Fitted	Resting facilitation
$\tau _D$	410 ms	Fitted	Recovery from desensitization
$H_D$	1.3 s$^{-1}$	Fitted	Desensitization decrement
EC50$_{AMPA}$	1000 $\mu$M	Featherstone and Shippy (2008)	AMPA receptor half-max
EC50$_{NMDA}$	4.7 $\mu$M	Featherstone and Shippy (2008)	NMDA receptor half-max
$Na^e$	130 mM	Cater et al. (2016)	Extracellular Na$^+$
$Na^a$	15 mM	Cater et al. (2016)	Astrocytic Na$^+$

The 1-compartment model shown for comparison in Fig. 5B (green), E (dotted lines) was obtained by setting $\gamma \rightarrow 0$ and $l \rightarrow 0$ in Eqs. (1–2), effectively collapsing the two-compartment description to a single isopotential neuron with $U=U_d$, receiving synaptic current $I_d$ directly at the soma. So, instead of Eqs. (1-3), the equation for *U*(*t*) becomes: $\tau _{m}~{dU}/{dt} = - \left( U - U_{L} \right) + {(I_{AMPA}+I_{NMDA}+I_{inj})}/{G_{s}}$.

The full set of parameters used for the simulation shown in Fig. 5 is given in Table 1.

The glutamate-mediated responses to the same stimulus, recorded in VC and CC modes and shown in Fig. 5A and B (black traces), reveal properties of current integration at the neuronal membrane and dendritic tree. Reconstructing the dendritic current (red in Fig. 5A) from the experimental somatic current (black) by means of Eq. (7) and then the dendritic and somatic membrane potentials (red dotted and solid, respectively, in Fig. 5B) by means of Eqs. (1,2), we fit the somatic voltage (Fig. 5B, red solid line) to the experimental voltage curve (Fig. 5B, black line) and thus obtain the parameters of the 2-compartment model of a neuron. The difference between the dendritic and somatic membrane potentials is caused by electrotonic effects at the dendritic tree, which re-shape the synaptic signals propagating towards soma. The 2-compartment model reproduces the shape well enough. In contrast, the 1-compartment model (green in Fig. 5B) gives the membrane potential that has different shape and bigger amplitude.

Responses from Fig. 5C are predominantly AMPAR-mediated currents in response to 5 stimuli, in the conditions with (black) and without (gray) blockade of glutamate transporters by TBOA. The contribution of NMDARs is small due to almost full magnesium block at the holding potential -80mV. The effect of TBOA is moderate, not changing the kinetics of each peak, but affecting the amplitude of the peaks, mostly the first ones. The second peaks are bigger, revealing facilitation. This synaptic plasticity is apparently of presynaptic origin. The model provides the following interpretation of these features. The amplification of the first peak in TBOA can be explained by the slight depolarisation of the presynaptic neuron by the elevated background glutamate concentration before the set of stimuli. Apparently, each peak’s shape is not affected by TBOA because it is mostly determined by the high amplitude peaks of the glutamate concentration in the center of the cleft. Only the peaks of the concentration but not the tails determine the course of AMPA responses, because AMPARs are not sensitive to low glutamate concentrations. The model (red and yellow) reproduces these features.

In the conditions of blocked AMPARs and at the holding voltage -20mV, we observe the response of NMDARs (Fig. 5D, black), which last much longer in the presence of TBOA (Fig. 5D, gray). The model accounts for this prolongation via slow clearance of glutamate from the perisynaptic space when EAAT-mediated uptake is removed (Eq. (13)). TBOA decreases the magnitude of the response, which we explain by the effect of partial desensitization of NMDARs by the glutamate remaining in the slice between series of stimulation. This background glutamate concentration is higher in the presence of TBOA; we denote it as $C_{glu}^0$ in the model, which we assume to be zero in the control case. The fraction of NMDARs activated by $C_{glu}^0$ is assumed to be desensitized and extracted from the total fraction of activated NMDARs according to Eq. (10). Consistently, $C_{glu}^{periph}$ diffuses down to the level of $C_{glu}^0$ in accordance to Eq. (13). The model (Fig. 5D, red and yellow) fits well to both experimental traces (Fig. 5D, black and gray), showing the contribution of the glutamate uptake in the form of the source term in Eq. (13), the diffusion that smoothes the glutamate concentration profile at the periphery of the cleft and the diffusion with the buffering extracellular solution in the extrasynaptic space.

Experimentally, currents obtained at two different voltages, $V_{h} = - 20mV$ and $V_{h} = + 40mV$, differ in shape (Fig. 5E, black and gray). One proposed explanation involves electrodiffusion (Savtchenko et al., 2017; Sylantyev et al., 2013): the electric field impedes the exit of negatively charged glutamate molecules from the cleft, which could explain why the response at $V_{h} = + 40mV$ (gray) is slower than at $V_{h} = - 20mV$ (black). However, the model reproduces this shape difference (red and blue, respectively) even without any voltage-dependence of diffusion, i.e., without electrodiffusion. In our model, the response shapes at $V_{h} = - 20mV$ and $V_{h} = + 40mV$ are different because of the electrotonic effects. This is an alternative mechanism to that proposed in Sylantyev et al. (2013).

Another significant effect of electrotonic propagation along the dendritic tree is seen by comparing the amplitudes of the responses at $V_{h} = - 20mV$ and $+ 40mV$ (black and gray in Fig. 5E). The amplitudes are quite similar, whereas they are quite different in 1-compartmental consideration (red and blue dashed lines in Fig. 5E). The responses obtained at different $V_{h}$, with the driving force stronger for the case of $V_{h} = + 40mV$ ($\left| V_{h} - V_{rev} \right| = 40mV$, assuming $V_{rev} \approx 0$) than for the case of $V_{h} = - 20mV$ ($\left| V_{h} - V_{rev} \right| = 20mV$). Because of this difference and because of the magnesium blockade, we could expect bigger response at $V_{h} = + 40mV$ (Fig. 5E, blue dotted line) than at $V_{h} = - 20mV$ (Fig. 5E, red dotted line). However, due to the space-clamp problem, the driving force at dendrite at $V_{h} = + 40mV$ is significantly lower than that at $V_{h} = - 20mV$, which compensates the effect of the magnesium block. The effect is reproduced in 2-compartmental model, where responses repeat the experimental curves (Fig. 5E, red and blue solid lines).

### Internal variables underlying the modelled responses

**Fig. 6:**
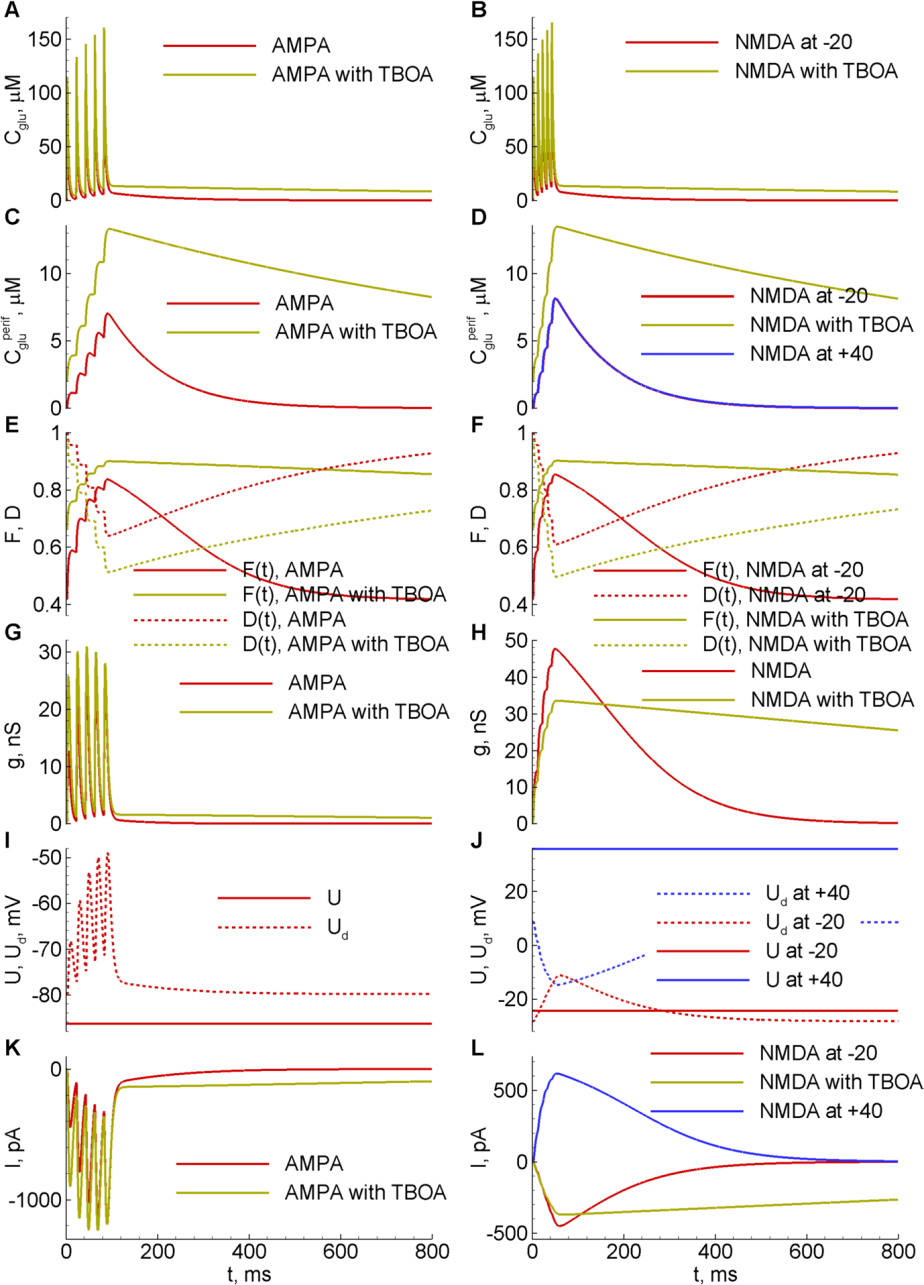
Internal variables underlying the modelled AMPA (left) and NMDA-mediated (right) responses. **A**-**D**, glutamate concentrations. **E**,**F**, synaptic facilitation and depression. **G**,**H**, synaptic conductances. **I**,**J**, somatic and dendritic membrane potentials. **K**,**L**, synaptic currents, the same as in Fig. 5

Whereas Fig. 5 shows experimentally observable variables, the quantities in Fig. 6 are model predictions that cannot be directly measured: glutamate concentrations, plasticity variables, and synaptic conductances. As the model predicts, the glutamate concentration in the cleft is in one order of magnitude greater than that on the periphery (compare Fig. 6A,B to Fig. 6C,D). At the later time stage, the concentrations converge. The steady-state level is elevated when the uptake is blocked. The glutamate concentration at the centre of the cleft is not much affected by TBOA as the glutamate concentration at the periphery, that is why the AMPA currents do not change so much in TBOA, whereas the NMDA currents do.

Synaptic plasticity exposed as different amplitudes of responses to the first and following stimuli in a series is seen in Fig. 5C. In the model, the synaptic plasticity is accounted for by the factors of facilitation and desensitization/depression, *F*(*t*) and *D*(*t*), in the r.h.s. of Eq. (12). The evolution of these factors is shown in Fig. 6E,F. The initial levels for *F*(*t*) are different in control (red) and TBOA (yellow) cases, due to its dependence (Eqs. (15)) on the glutamate concentration $C_{glu}^{periph}$, which, in turn, depends on the activity of EAATs. The model thereby explains the experimentally observed larger first peak under TBOA as a consequence of the elevated baseline $C_{glu}^{periph}$ when uptake is blocked. Regarding the dynamics of *F*(*t*), it is fully determined by the course of $C_{glu}^{periph}$ because of negligibly small $\tau _D$ in Eq. (15), as predicted by fitting, with the nonlinear dependence of *F*(*t*) on $C_{glu}^{periph}$ in the form of zeroed r.h.p. of that equation, which effectively reduces the order of the full system of ODEs from 7 to 6.

Consecutively, the glutamate concentrations $C_{glu}$ and $C_{glu}^{periph}$ determine the synaptic conductances (Fig. 6G,H). The shape of AMPA conductance (red in Fig. 6G) follows that of the glutamate concentration in the centre of the cleft (red in Fig. 6A); the shape of NMDA conductance (red in Fig. 6H) is determined by two factors: the concentration on the periphery (red and blue in Fig. 6D), and the voltage (red dashed in Fig. 6J) due to the magnesium block.

The somatic and dendritic voltages are different because of the space clamp problem (Fig. 6I,J). The dendritic voltage is close to the somatic one if the holding voltage is closer to the leak reversal potential, so the intercompartment voltage difference is more significant in the case of holding at +40mV than at -20mV (blue vs. red in Fig. 6J). In the case of NMDA, the shapes of dendritic potentials determine the effect of the magnesium block and result in different shapes of currents registered at -20 and +40mV (red and blue in Fig. 6L). In the case of AMPA, the shape of current (red in Fig. 6K) is similar to that of the conductance (red in Fig. 6G).

## Discussion

This study presents a mathematical model that successfully reproduces the kinetics of glutamatergic synaptic currents by incorporating glutamate transport, electrotonic effects, and synaptic plasticity. Formulated as a system of seven ordinary differential equations (ODEs), the model distinguishes between somatic and dendritic neuronal compartments while accounting for intrasynaptic and extrasynaptic glutamate diffusion. Our approach advances prior modeling efforts by integrating these key features into a computationally tractable framework that balances biological fidelity with scalability.

Unlike traditional synaptic models that treat conductance as a filtered spike train (Chizhov, 2014; Destexhe et al., 1994; Tranquillo & Neurophysiology, 2022), our model explicitly links glutamate dynamics to postsynaptic responses, re-interpreting the state variables in biophysical terms while retaining comparable mathematical complexity: the ODE system extends from 4th to 5th order for the conductance description alone, with the NMDA conductance following perisynaptic glutamate instantaneously and the AMPA conductance decaying with a delay set by the receptor closing time constant. This explicit linkage enables replication of the differential effects of EAAT2 blockade – where transporter inhibition prolongs NMDA receptor-mediated currents without altering AMPA kinetics – highlighting the critical role of astrocytic glutamate clearance. While spatially detailed models (Franks et al., 2002; Sylantyev et al., 2008) provide comprehensive descriptions of molecular diffusion, their computational demands limit applicability to network studies; by compartmentalizing spatial dynamics, our model achieves comparable predictive power while remaining efficient enough for studying pathological conditions as epilepsy (Zaitsev et al., 2020) or neurodegenerative diseases (Pajarillo et al., 2019).

An important direction for future work is to couple the present synaptic model with detailed multi-compartmental neuron models. To our knowledge, no established formulation currently exists that simultaneously describes glutamate concentration dynamics and the resulting synaptic conductances within a multi-compartmental framework; the standard practice in network simulators such as NEURON is to attach a reduced conductance kernel (alpha function or double-exponential) to one or a few compartments, bypassing mediator dynamics entirely. In contrast, our model provides a 7-variable ODE description of the full synaptic transmission chain (including glutamate dynamics, two receptor types, plasticity, and electrotonic propagation) that is suitable for network-scale modeling.

The model provides key insights into synaptic function. First, glutamate diffusion from the synaptic cleft to extrasynaptic spaces establishes concentration gradients that selectively influence receptor subtypes based on their distinct sensitivities. As glutamate rapidly exits the synaptic cleft, AMPA receptor responses cease abruptly, whereas NMDA receptor-mediated currents endure until transporters clear extrasynaptic glutamate. Second, electrotonic signal propagation along dendrites accounts for the comparable amplitudes of NMDA responses at -20 mV and +40 mV, presenting an alternative to mechanisms involving electrodiffusion (Sylantyev et al., 2013). Finally, the model’s predicted glutamate concentrations align with values derived from more intricate models (Gimenez et al., 2026; Sylantyev et al., 2008, 2013), despite the absence of direct experimental measurements, thereby supporting the validity of our simplified approach.

However, some questions remain unresolved. The model’s plasticity equations only partially account for the enhanced responses observed during EAAT2-dominant blockade, suggesting additional mechanisms may regulate presynaptic function under elevated glutamate conditions. This represents an important direction for future research.

While our model successfully captures the rapid dynamics of glutamate transport and receptor activation, it does not account for the long-term metabolic processes of glutamate recycling and astrocytic GABA release described in Flanagan et al. (2021). Their work demonstrates how elevated astrocytic glutamate levels can trigger GABA release via GAT-3 transporters, adding an inhibitory mechanism at glutamatergic synapses—a feature not included in our framework. This simplification allows our model to maintain computational efficiency for network-scale simulations while acknowledging that future extensions could incorporate such metabolic coupling to study pathologies like epilepsy, where glutamate-GABA crosstalk is critical. Similarly, the processes of longer time scales of neuro-glio-vascular coupling (Blanchard et al., 2016) and glutamate homeostasis (Shestov et al., 2012) are beyond the scope of our model.

One of the simplifications in the present model is the lumped implicit description of receptor desensitization and depression. AMPA receptors desensitize rapidly and profoundly in response to sustained glutamate exposure (Chen et al., 2002; Featherstone & Shippy, 2008), and this mechanism is relevant for understanding glutamate excitotoxicity under conditions of elevated extracellular glutamate. In the present experimental context—brief stimulation trains under well-buffered slice conditions—desensitization and depression effects are hardly distinguishable in the observed currents, and their full explicit description would not be supported by sufficient data for fitting. We note that the factor *D*(*t*) (Eq. (16)) captures some aspects of activity-dependent reduction in response amplitude, but it is mainly a postsynaptic receptor desensitization rather than a biophysical description of presynaptic depression. Future extensions of the model may include an explicit presynaptic depression for completeness and broader applicability.

The presynaptic facilitation model (Eq. (15)) is phenomenological in nature: rather than tracking vesicle pool dynamics, residual calcium, or spike timing explicitly, it uses perisynaptic glutamate concentration as a proxy signal for presynaptic state. This choice is motivated by the experimental observation that EAAT2-dominant blockade modifies the amplitude pattern of AMPA responses, suggesting that elevated extracellular glutamate feeds back onto presynaptic terminals via metabotropic glutamate receptors or extrasynaptic NMDA receptors (Chu & Hablitz, 2000; Duguid & Smart, 2009). While this approach does not fully capture the mechanistic detail of more biophysically constrained presynaptic models, it provides a compact and functionally adequate description that integrates naturally with the glutamate dynamics described by Eqs. (12–13).

Regarding the relationship of our model predictions to direct glutamate measurements: iGluSnFR-based optical glutamate sensors now allow real-time monitoring of extracellular glutamate dynamics with high spatial and temporal resolution (Hires et al., 2008). The spatiotemporal concentration profiles predicted by our model—with peak synaptic cleft concentrations on the order of tens of $\mu$M and perisynaptic concentrations of 1–3 $\mu$M—are broadly consistent with such measurements and with estimates from more detailed Monte Carlo models (Sylantyev et al., 2008, 2013). Future work could use iGluSnFR measurements to directly constrain and validate $\tau _{glu}$, $\tau _{diff}$, and $\beta$.

By combining experimental validation with computational efficiency, our model provides a useful tool for studying glutamatergic transmission in both physiological and pathological states. The explicit linkage between neurotransmitter dynamics, receptor activation, and dendritic integration offers new opportunities for investigating synaptic function at network scales while maintaining direct comparability to electrophysiological recordings. Overall, this study underscores the importance of glutamate transport and astrocytic involvement in maintaining synaptic homeostasis.

## Data Availability

No datasets were generated or analysed during the current study.
